# Landscape of the clinical development of China innovative anti-lung cancer drugs^☆^

**DOI:** 10.1016/j.cpt.2022.10.003

**Published:** 2022-10-11

**Authors:** Yuankai Shi

**Affiliations:** Department of Medical Oncology, National Cancer Center/National Clinical Research Center for Cancer/Cancer Hospital, Chinese Academy of Medical Sciences & Peking Union Medical College, Beijing Key Laboratory of Clinical Study on Anticancer Molecular Targeted Drugs, Beijing 100021, China

**Keywords:** Lung cancer, China, Anticancer drugs, Drug development

## Abstract

Even today, lung cancer remains one of the most frequently diagnosed cancers and the leading cause of cancer-related deaths worldwide. Throughout the past decades, remarkable advances have been made in the research and development of anti-lung cancer drugs in China. Since the first registered Chinese clinical trial on May 2, 2006, many potent anti-lung cancer drugs have been developed and approved by the China Food and Drug Administration and the National Medical Product Administration of China. Among them, the most advance were observed in the development of targeted agents and immunotherapeutic agents such as epidermal growth factor receptor (EGFR)-tyrosine kinase inhibitors (TKIs) icotinib, aumolertinib, and furmonertinib, anaplastic lymphoma kinase (ALK)-TKI ensartinib, programmed cell death-1 (PD-1) monoclonal antibodies (mAbs) camrelizumab, sintilimab, and tislelizumab, and programmed cell death-ligand 1 (PD-L1) mAb sugemalimab, which have made huge breakthrough in recent years. Some other investigational innovative drug also demonstrated promising efficacy and acceptable safety profiles. Results from clinical studies on these China innovative drugs have led to changes in clinical practice guidelines and considerably improved the outcomes for patients with lung cancer. Thus, in this review, we aim to provide further insight into the clinical development and achievement of China innovative anti-lung cancer drugs.

## Highlights

• China innovative anti-lung cancer drug clinical trials have increased greatly.
• Many China innovative targeted, immunotherapeutic, and antiangiogenic agents have been approved by China Food and Drug Administration/National Medical Product Administration.
• China innovative anti-lung cancer drugs significantly improved patients survival.

## Graphical abstract

**Figure:**
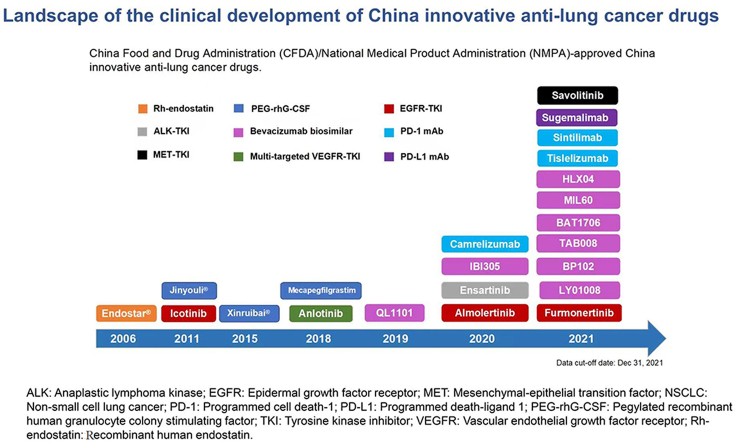

## Introduction

Cancer is one of the leading causes of death and a significant global health concern. The GLOBOCAN 2020 database illustrates the cancer-related public health burden within 185 countries and territories during 2020.^1^ Based on country-specific data obtained from 91 Chinese cancer registries, an estimated 4.569 million newly diagnosed cancer cases and 3.003 million cancer deaths occurred in China in 2020, accounting for 23.7% and 30.2% of all new cancer cases and cancer deaths worldwide, respectively.^1^^,^^2^ For this same year, lung cancer was both the most frequently diagnosed cancer and the leading cause of cancer-related deaths among all cancer types, with an estimated 0.816 million lung cancer diagnoses and 0.715 million deaths in China.^1^^,^^2^ In recent years, multiple anti-lung cancer drugs have been developed and approved domestically, providing tremendous benefits to Chinese patients with lung cancer.^3^ Thus, we present this review to provide additional insight into the clinical development of China innovative anti-lung cancer drugs.

### Survival outcome of lung cancer

Lung cancer is considered a heterogenous disease; where according to the histology presentation, it can be classified into non-small cell lung cancer (NSCLC) and small cell lung cancer (SCLC), accounting for approximately 85% and 15% of all lung cancer cases, respectively.^4^ Among NSCLC, lung adenocarcinoma is the most common subtype, followed by lung squamous cell carcinoma.^5^ Between 2005 and 2014, the proportion of lung adenocarcinoma diagnoses among 7184 lung cancer patients from seven geographic regions of China increased from 36.4% to 53.5% (*P* ​< ​0.001), while that of squamous cell carcinoma decreased from 45.4% to 34.4% (*P* ​< ​0.001). Of these cases, the proportions of stages I–IV lung cancer were approximately 19%, 16.5%, 34.7%, and 29.9%, respectively.^6^ Another study revealed that the five-year overall survival (OS) rate for lung cancer in Shanghai, China, declined from 55.5% for stage I patients to 5.3% for stage IV patients.^7^ Furthermore, Zeng et al.^8^ described the changing cancer survival rate in China between 2003 and 2015, where the five-year OS rate for all cancers improved by 9.6% (from 30.9% [2003–2005] to 40.5% [2012–2015]), while that for lung cancer exhibited only a slight increase of 3.6% (from 16.1% [2003–2005] to 19.7% [2012–2015]). One of the main explanations for this stunted improvement in survival is that many patients with lung cancer were initially diagnosed in the advanced stage. Our previous multicenter prospective study reported that among 1324 patients with unresectable stage IIIb/IV non-small cell lung cancer in China, the median OS was 23.2 months (95% confidence interval [CI] 19.5–25.5), where the one-year and three-year OS rates were 68.9% and 39.0%, respectively. Furthermore, patients treated in Tier-1 cities (Beijing and Shanghai) exhibited better OS outcomes than those in Tier-2 cities (Harbin, Changchun, Xi'an, Wuhan, Chengdu, Chongqing, Fuzhou, and Nanning) (35.4 *vs.* 16.3 months, *P* ​< ​0.001).^9^

### Clinical trials on lung cancer

With the advent of the 21st century, the development of anticancer drug clinical trials in China has entered a new era. The guidance and encouragement of national policies have promoted extensive collaboration between multiple sectors and robustly boosted the research and development of new anticancer drug pipelines in China. Additionally, significant progress and breakthroughs have been made over recent years, promoting the shift from imitation to original innovation.^10^, ^11^, ^12^, ^13^

The first anticancer drug clinical trial was registered with the Chinese Clinical Trial Registry (ChiCTR) platform (http://www.chictr.org.cn) on May 2, 2006. As of April 1, 2022, a total of 1974 hospitals have been approved by the National Medical Product Administration (NMPA) of China as national drug clinical trial sites, among which 894 belonged to anticancer drug clinical trial sites, according to the data on the NMPA official website (http://app1.nmpa.gov.cn/). It should be noted that NMPA was named as the China Food and Drug Administration (CFDA) before 2018. Additionally, 17,111 clinical trials have been registered between January 1, 2012, and June 30, 2022, on the Chinese Drug Clinical Trial Registration and Information Publicity platform (http://www.chinadrugtrials.org.cn). From 2010 to 2020, among 1410 drugs with approved first investigational new drug (IND) applications in China, 705 drugs (50%) have reached at least phase I clinical trials, 286 drugs (20%) have reached phase II, and 108 drugs (8%) have reached phase III. Interestingly, anticancer drugs comprise the largest proportion of all innovative drugs.^14^

We previously reported the recent changes in the anti-lung cancer clinical trial landscape within the mainland of China.^15^ From 2005 to 2013, few clinical trials on lung cancer were conducted, while since 2013, the number of clinical trials has increased significantly, with an annual growth rate of 26.5%. Overall, among 1595 clinical trials of anti-lung cancer drugs, 698 (43.8%) and 267 (16.7%) clinical trials were sponsored by domestic and international industries, respectively, and the remaining 630 (39.5%) clinical trials were investigator-initiated trials (IITs). Among industry-sponsored trials (ISTs), phase III clinical trials accounted for 32.1% of ISTs, followed by phase II at 20.3%, phase I at 24.5%, phase IV at 7.2%, and phase I/II at 7.0%. Conversely, in IITs, phase II clinical trials comprised the leading proportion at 44.8%. Within targeted drug ISTs, trials involving epidermal growth factor receptor (EGFR)-tyrosine kinase inhibitors (TKIs), vascular endothelial growth factor receptor (VEGFR)-TKIs, and anaplastic lymphoma kinase (ALK)/ROS proto-oncogene 1 (ROS1)/mesenchymal–epithelial transition factor (MET)-TKIs accounted for 38.3%, 17.0%, and 16.3% of the total trials, respectively. In immunotherapeutic agent trials, programmed cell death-1 (PD-1) and programmed cell death-ligand 1 (PD-L1) monoclonal antibodies (mAbs) were predominantly investigated with proportions of 44.0% and 21.1%, respectively.^15^

### Recent advances in anti-lung cancer drug clinical development

**Figure 1:**
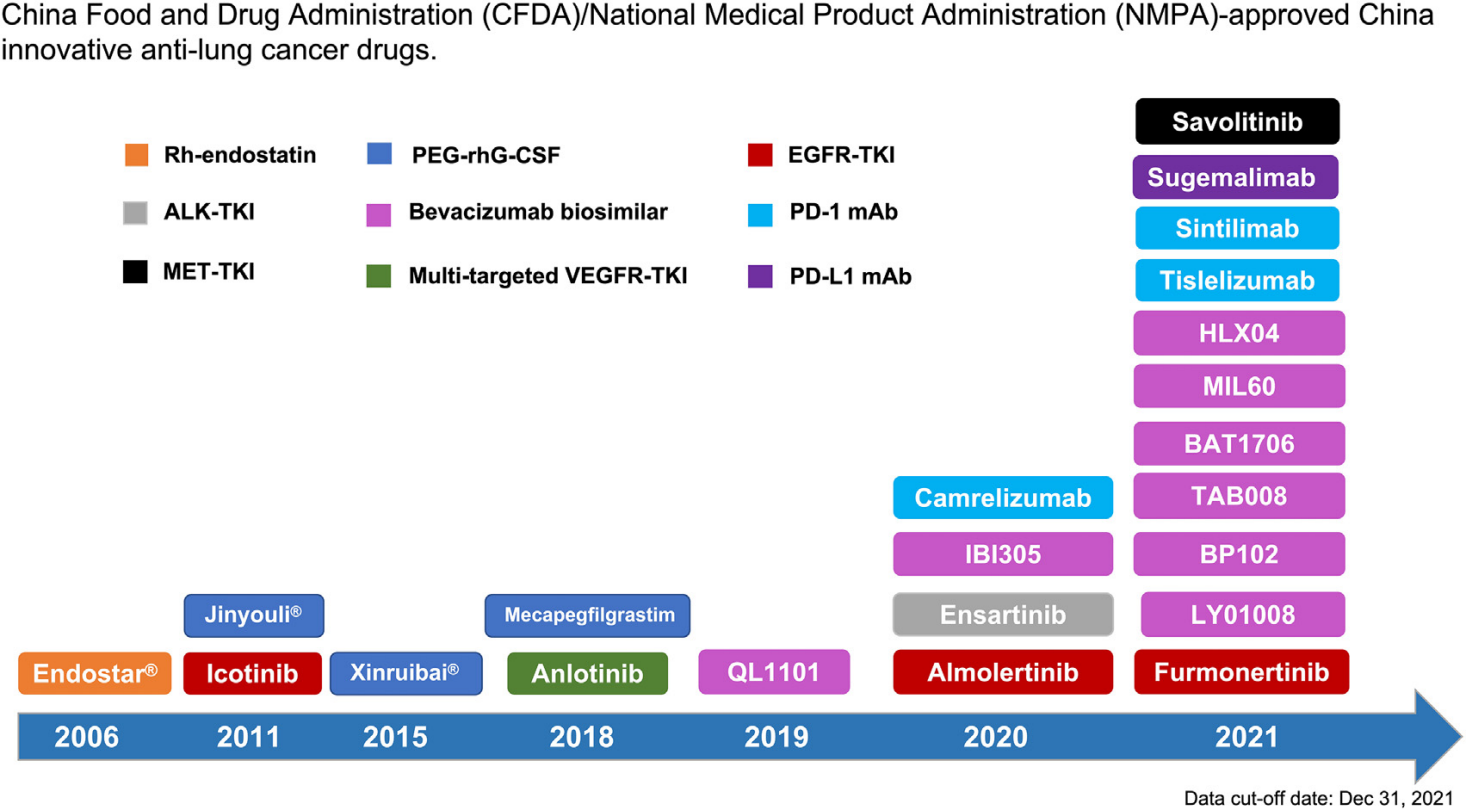
China Food and Drug Administration (CFDA)/National Medical Product Administration (NMPA)-approved China innovative anti-lung cancer drugs. ALK: Anaplastic lymphoma kinase; EGFR: Epidermal growth factor receptor; MET: Mesenchymal-epithelial transition factor; NSCLC: Non-small cell lung cancer; PD-1: Programmed cell death-1; PD-L1: Programmed death-ligand 1; PEG-rhG-CSF: Pegylated recombinant human granulocyte colony stimulating factor; TKI: Tyrosine kinase inhibitor; VEGFR: Vascular endothelial growth factor receptor; Rh-endostatin: Recombinant human endostatin; mAb: Monoclonal antibody.

Several new innovative drugs or biosimilars related to targeted therapy, immunotherapy, antiangiogenesis therapy, and supportive care have been developed during the past two decades in China, significantly contributing to medical care advances for patients with lung cancer and other cancers. The current CFDA/NMPA-approved China innovative anti-lung cancer drugs are listed in Table 1 and Figure 1.

**Table 1:** China Food and Drug Administration (CFDA)/National Medical Product Administration (NMPA)-approved China innovative anti-lung cancer drugs.

Class	Drug name	Application	Approved date
Anti-angiogenetic agent	Rh-endostatin (Endostar®)	Combined with vinorelbine and cisplatin for locally advanced or metastatic NSCLC	July 23, 2006
PEG-rhG-CSF	Pegylated filgrastim (Jinyouli®)	Febrile neutropenia	Oct 17, 2011
	Pegylated filgrastim (Xinruibai®)	Febrile neutropenia	Aug 26, 2015
	Mecapegfilgrastim	Febrile neutropenia	May 8, 2018
EGFR-TKI	Icotinib	≥2L NSCLC	June 17, 2011
		1L advanced EGFR-sensitizing mutant NSCLC	Nov 13, 2014
	Almonertinib	Advanced EGFR-T790M NSCLC after progression on first- and second-generation EGFR-TKIs	March 18, 2020
		1L EGFR-sensitizing mutated NSCLC	Dec 4, 2021
	Furmonertinib	Advanced EGFR-T790M NSCLC after progression on first- and second-generation EGFR-TKIs	March 3, 2021
		1L EGFR-sensitizing mutated NSCLC	June 28, 2022
ALK-TKI	Ensartinib	2L ALK-positive NSCLC	Nov 19, 2020
		1L ALK-positive NSCLC	Mar 18, 2022
MET-TKI	Savolitinib	Locally advanced and metastatic METex14+ NSCLC	June 22, 2021
VEGFR-associated multi-targeted TKIs	Anlotinib	≥3L NSCLC	May 9, 2018
		≥3L SCLC	Sept 4, 2019
Bevacizumab biosimilar	QL1101	1L combined with chemotherapy for advanced or recurrent non-squamous NSCLC	Dec 9, 2019
	IBI305	Same as above	June 19, 2020
	LY01008	Same as above	May 7, 2021
	BP102	Same as above	June 22, 2021
	BAT1706	Same as above	Nov 19, 2021
	MIL60	Same as above	Nov 26, 2021
	TAB008	Same as above	Dec 1, 2021
	HLX04	Same as above	Dec 3, 2021
PD-1 mAb	Camrelizumab	1L EGFR/ALK-negative non-squamous NSCLC	June 19, 2020
		1L squamous NSCLC	Dec 10, 2021
	Sintilimab	1L EGFR/ALK-negative non-squamous NSCLC	Feb 3, 2021
		1L squamous NSCLC	June 3, 2021
	Tislelizumab	1L EGFR/ALK-negative non-squamous NSCLC	June 22, 2021
		1L squamous NSCLC	Jan 14, 2021
PD-L1 mAb	Sugemalimab	1L EGFR/ALK-negative NSCLC	Dec 21, 2021

ALK: Anaplastic lymphoma kinase; EGFR: Epidermal growth factor receptor; MET: Mesenchymal-epithelial transition factor; NSCLC: Non-small cell lung cancer; PD-1: Programmed cell death-1; PD-L1: Programmed death-ligand 1; PEG-rhG-CSF: Pegylated recombinant human granulocyte colony stimulating factor; TKI: Tyrosine kinase inhibitor; VEGFR: Vascular endothelial growth factor receptor; mAb: Monoclonal antibody; Rh-endostatin: Recombinant human endostatin.

### Targeted therapy

Throughout the past 20 years, various effective small molecule TKIs have been developed for lung cancer treatment, including EGFR/ALK/ROS-1/MET/VEGFR/Human epidermal growth factor receptor 2 (HER2)/Kirsten rat sarcoma viral oncogene (KRAS)/RET proto-oncogene (RET)/neurotrophin receptor kinase (NTRK)/v-raf murine sarcoma viral oncogene homolog B1 (BRAF)/mitogen-activated protein kinase (MEK)/fibroblast growth factor receptor (FGFR)-TKIs. These discoveries have shifted the focus toward targetable genetic alterations for the treatment of multiple solid tumors, especially in lung cancer.^16^

### Epidermal growth factor receptor-tyrosine kinase inhibitor (EGFR-TKI)

EGFR-sensitizing mutations are the most common targetable driver mutations observed in lung adenocarcinoma. Their prevalence varies in different geographical regions, ranging from 5 to 15% in Caucasian to 40–55% in Asia.^17^, ^18^, ^19^ From 2005 to 2020, 275 EGFR-TKIs clinical trials were conducted in the mainland of China.^15^ Three generations of EGFR-TKIs are currently available on a commercial scale.^20^, ^21^, ^22^, ^23^, ^24^, ^25^, ^26^, ^27^ Of these, important EGFR-TKIs include gefitinib, erlotinib, icotinib, afatinib, dacomitinib, osimertinib, aumolertinib, and furmonertinib. Gefitinib served as a first-generation EGFR-TKI, and compared with traditional chemotherapy, the drug significantly prolonged the survival of patients with lung adenocarcinoma harboring the EGFR-sensitizing mutation.^20^ Furthermore, its development initiated the era of targeted lung cancer therapy worldwide. Icotinib was the first EGFR-TKI developed in China.^28^ Specifically, it was presented as a second or third line of treatment for patients with advanced NSCLC who had pretreated with chemotherapy. In the ICOGEN phase III clinical trial for icotinib, the drug demonstrated non-inferior anticancer efficacy compared with gefitinib (median progression-free survival [mPFS] 4.6 *vs.* 3.4 months, HR = 0.84, *P* = 0.13), along with fewer treatment-related adverse events (61% *vs.* 70%, *P* ​= ​0.046).^29^ Given these results, icotinib was approved by the CFDA on June 17, 2011, and awarded the 2015 National Science and Technology Progress Award, first prize. A comment article in The Lancet Oncology entitled “Icotinib: kick-starting the Chinese anticancer drug industry” stated that icotinib “might herald a wave of new anticancer drugs, rapidly and cheaply developed in China before going on to penetrate other world markets”.^30^ Afterward, the results of CONVINCE phase III clinical trial established icotinib as a front-line treatment for patients with EGFR-sensitizing mutations in lung adenocarcinoma, where the drug prolonged PFS to 11.2 months (compared with 7.9 months associated with chemotherapy HR = 0.61, [*P* = 0.006]) and also decreased the prevalence of drug-related adverse events by 36.4% compared with that by chemotherapy (54.1% *vs.* 90.5%, *P* ​< ​0.001).^22^ Additionally, several additional clinical trials with icotinib have been carried out to expand its application.^31^^,^^32^

A total of 40–55% of the resistance to first- and second-generation EGFR-TKIs is because of the acquired *EGFR* Thr790Met (T790M) mutation.^33^, ^34^, ^35^ Osimertinib is a third-generation EGFR-TKI targeting both EGFR-sensitizing and *EGFR* T790M mutations.^36^ Two third-generation EGFR-TKIs, aumolertinib and furmonertinib, were developed in China and subsequently approved by the NMPA on March 18, 2020 and March 3, 2021 for the treatment of advanced primary *EGFR* T790M-positive NSCLC and to supplement first- and second-generation EGFR-TKIs.^37^^,^^38^ Aumolertinib and furmonertinib exhibited objective response rates (ORRs) of 68.9% and 73.6%, respectively, for the previously mentioned patient populations. Furthermore, they both displayed favorable blood–brain penetration and activities in the central nervous system (CNS). Subsequently, aumolertinib and furmonertinib were also approved as first-line treatments for EGFR-mutated NSCLC on December 4, 2021, and June 28, 2022, respectively. Additionally, the PFS of aumolertinib was significantly extended compared with that of gefitinib (19.3 *vs.* 9.9 months, hazard ratio [HR] ​= ​0.46, *P* < 0.0001) in treatment-naïve EGFR-sensitizing-mutated NSCLC.^26^ The first-line results of furmonertinib indicated that it produced a significantly longer PFS (20.8 *vs.* 11.1 months, HR ​= ​0.44, *P* < 0.0001) compared with gefitinib.^27^ For patients with CNS metastasis, furmonertinib also demonstrated favorable efficacy.^39^ Several other domestically developed third-generation EGFR-TKIs such as rezivertinib (BPI7711), limertinib (ASK120067), befotertinib (D-0316), and oritinib (SH-1028) have also shown favorable efficacy and safety among patients with NSCLC showing *EGFR* T790M mutations.^40^, ^41^, ^42^, ^43^, ^44^

The EGFR exon 20 insertion (EGFR exon20ins) mutation appeared in approximately 2% of the NSCLC patients and unfortunately responded poorly to first- and second-generation EGFR-TKIs.^45^ In response to the phase I/II clinical trial for mobocertinib, where it displayed an ORR of 28%, mobocertinib was approved by the U.S. Food and Drug Administration (FDA) for patients with NSCLC harboring EGFR exon20ins mutation on Sep 15, 2021.^46^ Furthermore, the EGFR and MET bispecific antibody (BsAb) amivantamab received an ORR of 40% in EGFR exon20ins-driven NSCLC.^47^ However, there is still scope for improving the treatment dilemma caused by this specific mutation, and promising studies are already underway. Recently, an innovative, Chinese-developed selective EGFR-TKI named sunvozertinib (DZD9008) showed promising activity against EGFR exon20ins mutations in its preclinical and phase I clinical trials, boasting an ORR of 37.5% and manageable toxicity.^48^

### *Anaplastic lymphoma kinase*/*ROS proto-oncogene 1-tyrosine kinase inhibitor (ALK/ROS1-TKI)*

ALK gene rearrangement occurs in 3–5% of the NSCLC patients and is known for the remarkable treatment benefits it provides alongside ALK-TKIs.^49^^,^^50^ Crizotinib is the only commercially available first-generation ALK-TKI and has been followed by several second-generation ALK-TKIs such as alectinib, ceritinib, brigatinib, and ensartinib, which exhibit improved selectivity and CNS penetration.^51^, ^52^, ^53^, ^54^, ^55^ Following the phase III clinical trial for alectinib, results showed that the drug significantly prolonged mPFS in patients with advanced untreated ALK-positive NSCLC, more so than did crizotinib (34.8 *vs.* 10.9 months, HR ​= ​0.43 [95% CI 0.32–0.58]), alectinib has been approved by the FDA and NMPA as a first-line treatment option.^52^^,^^56^ Ensartinib is a domestically developed ALK-TKI that was approved by the NMPA on November 19, 2020, for the treatment of locally advanced or metastatic ALK-positive NSCLC as a successive treatment after crizotinib or for crizotinib-intolerant patients.^57^ The phase II clinical trial for ensartinib reported an ORR of 52% and a manageable safety profile among patients progressing on crizotinib.^55^ Two other second-generation ALK-TKIs, ceritinib and brigatinib, were approved by the NMPA on May 31, 2018, and Mar 24, 2022, respectively.^54^^,^^58^ Most recently, the third-generation ALK-TKI lorlatinib was approved by the NMPA on April 30, 2022.^59^

ROS1 rearrangements are observed in 1–2% of the NSCLC patients.^60^ Because they share high structure homology with ALK, many ALK-TKIs such as crizotinib, ceritinib, and lorlatinib also exhibited activity against ROS1. With an ORR of 72%, crizotinib was approved by the CFDA for patients with advanced ROS1-positive NSCLC on October 17, 2017.^61^ Entrectinib, another first-line standard treatment for advanced ROS1-positive NSCLC, was approved by the NMPA on August 12, 2022.^62^ On July 29, 2022, the NMPA also approved entrectinib for NTRK-positive solid tumors.^63^ Iruplinalkib (WX-0593) is a Chinese innovative ALK/ROS1-TKI, which has shown promising anticancer activity and favorable safety profile among patients with advanced NSCLC harbouring ALK or ROS1 rearrangement. In its phase I clinical trial, iruplinalkib was associated with ORRs of 81.0% and 76.3% in patients with treatment-naïve ALK-positive NSCLC in the dose-escalation and -expansion phases, respectively, and with ORRs of 38.1% and 45.7% in patients who had previously received crizotinib in the dose-escalation and -expansion phases, respectively.^64^ In its phase II clinical trial involving 146 patients with ALK-positive crizotinib-resistant NSCLC, ORR for iruplinalkib was 69.9%.^65^ Its phase III (NCT04632758) clinical trial is ongoing.

### Mesenchymal-epithelial transition factor-tyrosine kinase inhibitor (MET-TKI)

MET exon14 skipping (METex14) mutations occur in approximately 3–4% of the NSCLC patients and 20–32% of the pulmonary sarcomatoid carcinoma patients.^66^^,^^67^ Selective MET-TKIs such as savolitinib, capmatinib, and tepotinib have emerged in recent years and showed considerable progress in patients with METex14 mutations or MET amplification.^68^, ^69^, ^70^ Based on the results of its phase II clinical trial, which reported an ORR of 49.2% and an acceptable safety profile, savolitinib was approved on June 22, 2021, by the NMPA as the first MET-TKI intended for the treatment of patients with locally advanced and metastatic METex14-positive NSCLC in China.^68^ Another MET-TKI, glumetinib, received an ORR of 60.9% among patients with METex14-positive NSCLC, increasing its possibility as a new treatment option in the near future.^71^

### Human epidermal growth factor receptor 2-tyrosine kinase inhibitor (HER2-TKI)

Human epidermal growth factor receptor 2 (HER2) is a member of the ErbB family. Notably, HER2 gene mutations, gene amplification, and protein overexpression were reported in 1–4%, 2–5%, and 2–30% of the NSCLC patients, respectively.^72^ For these patients with HER2-positive advanced NSCLC, chemotherapy remains the first-line choice of treatment. Pyrotinib, a novel pan-ErbB inhibitor developed in China, showed promising anticancer activity in both a preclinical study and phase II clinical trial in patients with HER2-mutated NSCLC, who had previously undergone chemotherapy, exhibiting an ORR of 30–53%, and a manageable safety profile.^73^^,^^74^ Some antibody–drug conjugates (ADCs) such as trastuzumab-emtansine (T-DM1) and trastuzumab-deruxtecan (T-Dxd, DS-8201) have also displayed encouraging activities against HER2-overexpressing and HER2-mutated NSCLC, with ORRs of 20–55%.^75^^,^^76^

### Other tyrosine kinase inhibitors (TKIs)

Apart from the targets mentioned previously, there are additional important driving alterations of NSCLC, such as KRAS/RET/NTRK/BRAF/MEK/FGFR. For example, KRAS mutation was observed in approximately 35% of lung adenocarcinomas and 5% of squamous NSCLC patients.^77^ Previously, KRAS was considered an untargeted mutation and associated with poor response to platinum-based chemotherapy during the treatment of advanced NSCLC, making it a challenge for clinicians.^78^ On May 28, 2021, the KRAS-TKI sotorasib was successfully developed and approved by the FDA for the treatment of KRAS G12C-positive NSCLC in those having received at least one previous systemic therapy, exhibiting an ORR of 36% and disease control rate (DCR) of 81% in these patients.^79^^,^^80^ Several other TKIs have also made global progress, some of which have been approved by the NMPA (Supplementary Table 1).^63^^,^^81^, ^82^, ^83^, ^84^, ^85^, ^86^ Novel TKIs related to the previously mentioned targets are currently under clinical investigation in China, with the hope that they can provide new choices for patients with NSCLC in the future.

### Anti-angiogenesis agents

Angiogenesis plays a key role in tumorigenesis and metastasis. Since the great breakthrough in anticancer angiogenesis theory in the 1970s,^87^ agents related to anti-angiogenesis have become a research hotspot. Recombinant human (rh)-endostatin (Endostar®) is a China innovative anti-angiogenetic agent and was approved on July 23, 2006, by the CFDA for patients with treatment-naïve or previously treated stage III–IV NSCLC in combination with vinorelbine and cisplatin.^88^ For this, rh-endostatin was awarded the 2008 National Technological Innovation Award, second prize. VEGFR-associated multi-targeted TKI anlotinib has achieved commendable success in the treatment of both NSCLC and SCLC. Anlotinib was approved by the NMPA on May 9, 2018, for locally advanced or metastatic NSCLC, which had progressed or relapsed after at least two lines of systematic chemotherapy,^89^ and for the same condition in SCLC on September 4, 2019.^90^

Apart from rh-endostatin and small molecular TKIs, several antibodies have also demonstrated potent activities against various cancers, including lung cancer. Bevacizumab (Avastin®) is a VEGFR mAb that is an important treatment for advanced or recurrent nonsquamous NSCLC. Clinical development of bevacizumab biosimilars has made progress in China, with eight biosimilars, including QL1101, IBI305, LY01008, MIL60, TAB008, BP102, BAT1706, and HLX04, having been approved by the NMPA since December 9, 2019. These biosimilars were approved based on the results of phase III clinical trials comparing their efficacy, safety, and immunogenicity with that of bevacizumab (Avastin®) when used in combination with chemotherapy as the first-line treatment for patients with advanced or recurrent nonsquamous NSCLC (except for HLX04).^91^, ^92^, ^93^, ^94^, ^95^, ^96^, ^97^ For example, in its phase III clinical trial, LY01008, in combination with paclitaxel and carboplatin, demonstrated similar efficacy (ORR: 48.5% *vs.* 53.0%, the stratified ORR ratio ​= ​0.91 [90% CI 0.80–1.04]) to the bevacizumab (Avastin®) group, supporting the clinical equivalence of the two agents (0.75–1.33).^93^ Furthermore, the safety and immunogenicity profiles of LY01008 and bevacizumab (Avastin®) were also similar.

### Immunotherapy

Unlike traditional chemotherapy and targeted therapies, immunotherapy, especially immune checkpoint inhibitors (ICIs), mark a shift from enhancement to normalization of the human immune system.^98^ This has changed the treatment modality of various types of malignant tumors and has become a new milestone in medical oncology. PD-1/PD-L1/cytotoxic T-lymphocyte-associated protein 4 (CTLA-4) are currently the most widely investigated and applied ICIs. Since the approval of the PD-1 mAb, pembrolizumab, for previously treated melanoma by the FDA on September 4, 2014, multiple ICIs have followed suit and joined the wave of clinical development. Thus far, many innovative PD-1/PD-L1 mAbs have been successfully developed in China.

In the treatment of NSCLC, three PD-1 mAbs have been developed by different domestic industries: camrelizumab,^99^^,^^100^ sintilimab,^101^^,^^102^ and tislelizumab,^103^^,^^104^ which have all been approved by the NPMA as the first-line treatment of both EGFR/ALK-negative nonsquamous and squamous NSCLC in combination with chemotherapy, regardless of PD-L1 expression. Clinical trials have reported that these drugs prolong the PFS by 2–4 months and increase the ORR by approximately 20%, while the improvement in OS is still immature. The phase III clinical trials of toripalimab, another PD-1 antibody, as a first-line treatment for advanced NSCLC have also shown promising results.^105^ PD-L1 mAb sugemalimab was initially approved by NMPA for the same condition, based on the results of the GEMSTONE-302 study with nonsquamous and squamous NSCLC cohorts.^106^ Supplementing chemotherapy with PD-1/PD-L1 mAbs has significantly improved the efficacy of front-line treatment for advanced NSCLC as compared with chemotherapy alone and also demonstrated a manageable safety profile. Meanwhile, in second and third-line settings, sintilimab and tislelizumab have also achieved positive results. In the ORIENT-3 study, sintilimab was associated with longer PFS (4.3 *vs.* 2.8 months, HR = 0.52, *P* < 0.0001) and OS (11.8 *vs.* 8.3 months, HR = 0.74, *P* = 0.025) in previously treated advanced/metastatic squamous NSCLC, as compared with docetaxel.^107^ The RATIONALE-303 study compared tislelizumab to docetaxel as second/third-line therapies in patients with advanced NSCLC achieved and found that tislelizumab prolonged the PFS (4.1 *vs.* 2.6 months, HR = 0.64, *P* < 0.0001) and OS (17.2 *vs.* 11.9 months, HR = 0.64, *P* < 0.0001), regardless of histology or PD-L1 expression.^108^ Additionally, the PD-L1 mAb, envafolimab, has been approved for subcutaneous administration to treat solid tumors with defective mismatch repair (dMMR)/microsatellite instability-high (MSI-H); however, the clinical trial included mainly gastrointestinal cancer as opposed to lung cancers.^109^

In the treatment of SCLC, combining PD-L1 mAb atezolizumab/durvalumab with etoposide plus platinum (EP) chemotherapy has become the standard treatment for extensive-stage SCLC, prolonging OS by 2–3 months.^110^^,^^111^ Recently, in patients with extensive-stage SCLC, compounding innovative Chinese PD-L1 mAb, adebrelimab, with EP chemotherapy has also shown better efficacy than EP chemotherapy alone, extending OS (15.3 *vs.* 12.8 months, HR = 0.72, *P* = 0.0017) and PFS (5.8 vs. 5.6 months, HR = 0.67, *P* < 0.0001) and yielding manageable toxicity.^112^ PD-1 mAb serplulimab plus chemotherapy also significantly improved OS of treatment-naïve extensive-stage SCLC.^113^

Apart from combination with chemotherapy, attempts have also been made to explore the efficacy and safety of combining ICIs with antiangiogenetic agents. A phase Ib clinical trial indicated the efficacy of sintilimab in conjunction with anlotinib as first-line therapy for advanced NSCLC, producing an ORR of 72.7%.^114^ In another phase III clinical trial, sintilimab combined with IBI305 (a bevacizumab biosimilar) and chemotherapy significantly improved PFS among patients with EGFR-mutated nonsquamous NSCLC, which progressed after EGFR-TKI treatment, as compared with chemotherapy alone (6.9 *vs.* 4.3 months, HR = 0.46, *P* < 0.0001).^115^ Moreover, some novel targets for immunotherapy, such as lymphocyte-activation gene 3 (LAG-3), T cell immunoglobulin (Ig) and mucin protein 3 (TIM-3), T cell immunoreceptor with Ig and immunoreceptor tyrosine-based inhibitory motif domains (TIGIT), and CD47, are also being investigated worldwide as either independent treatments or together with other ICIs.^116^, ^117^, ^118^, ^119^

### Bispecific antibody (BsAb) and antibody-drug conjugate (ADC)

BsAbs and ADCs are two novel classes of agents in the treatment pipeline of lung cancer. BsAbs are a large family of molecules that specifically bind to two different epitopes or antigens, thus strengthening specificity and reducing toxicity.^120^ Many novel Chinese BsAbs, such as anti–CTLA-4/PD-1 inhibitors AK104, SI–B003, and KN046, are being clinically investigated in lung cancer. The results are being awaited.

ADCs are composed of a highly selective mAb, a potent cytotoxic agent, and a linker, giving it the advantages of both specific targeting and direct cell-killing.^121^ Rovalpituzumab tesirine (Rova-T) is the first ADC targeting delta-like protein 3 (DLL3) in SCLC. However, its phase III clinical trial demonstrated only modest clinical activity in third-line settings and beyond in SCLC, with an ORR of 12.4%.^122^ Telisotuzumab vedotin (ABBV-399) is an ADC targeting MET, but its phase II clinical trial on MET-positive squamous advanced and recurrent NSCLC failed to meet the pre-specified response, with an ORR of only 9% and unanticipated toxicity causing pneumonia.^123^

### Pegylated recombinant human granulocyte colony-stimulating factor (PEG-rhG-CSF)

Neutropenia is one of the most common chemotherapy toxicities, increasing the risk of infection and death, hindering the administration of chemotherapy under the planned dose, and dampening the therapeutic effect.^124^ Compared with recombinant human granulocyte colony-stimulating factor (rhG-CSF), pegylated recombinant human granulocyte colony-stimulating factor (PEG-rhG-CSF) is modified by polyethylene glycol and possesses decreased immunogenicity and a significantly longer half-life, resulting in reduced injection frequency and improved patient compliance.^125^^,^^126^ On August 26, 2011, pegylated filgrastim (Jinyouli®) became the first marketed China innovative PEG-rhG-CSF approved by CFDA for patients with cancer and febrile neutropenia who underwent anticancer treatment. A phase III clinical trial confirmed that the incidences of febrile neutropenia and subsequent antibiotic administration were similar in both the pegylated filgrastim (Jinyouli®) and rhG-CSF (Jinxuli®) groups.^127^ The study “Establishment and industrialization of key technical system of recombinant protein drugs modified by polyethylene glycol” was awarded the 2020 National Science and Technology Progress Award, second prize. Subsequently, two more PEG-rhG-CSFs were approved by the NMPA [Table 1].

## Conclusion

In recent years, China innovative anticancer drug clinical trials have increased greatly. Various China innovative targeted agents and immunotherapeutic agents have been approved by the NMPA, including EGFR-TKIs, ALK-TKIs, PD-1/PD-L1 mAbs, and many of these are now included in the National Healthcare Security Administration (NHSA) medical insurance list. Clinical study results for these China innovative drugs have changed clinical practice guidelines and improved the clinical outcomes for patients with lung cancer drastically.^128^, ^129^, ^130^ In the future, more China innovative drugs will bring more survival benefit for patients with lung cancer.

## Funding

This work was supported by the China National Major Project for New Drug Innovation (No. 2017ZX09304015).

## Author contribution

Yuankai Shi: Conceptualization, investigation, data curation, writing-original draft preparation, writing-reviewing, and editing.

## Ethics statement

None.

## Data availability statement

The datasets used in the current study are available from the corresponding author on reasonable request.

## Conflict of interest

None.
