# Supplementary material for: Landscape of the clinical development of China innovative anti-lung cancer drugs
Source: Cancer Pathog Ther. 2022 Oct 11;1(1):67–75. doi: 10.1016/j.cpt.2022.10.003 (PMC10846302; doi:10.1016/j.cpt.2022.10.003)
Supplement: Multimedia component 1 [file mmc1.docx]

**Supplementary Table 1. Kirsten rat sarcoma viral oncogene (KRAS)/RET proto-oncogene (RET)/Neurotrophin receptor kinase (NTRK)/B-raf murine sarcoma viral oncogene homolog B1 (BRAF)/Mitogen-activated protein kinase (MEK)/Fibroblast growth factor receptor-Tyrosine kinase inhibitor (FGFR-TKIs).**

| **Class** | **Drug name** | **Application** | **Date on the market** |
| --- | --- | --- | --- |
| *KRAS* | Sotorasib | 2L KRAS G12C-positive NSCLC | Approved on May 28, 2021 by the U.S. FDA |
| *RET* | Pralsetinib | RET-positive NSCLC | Approved on Sept 4, 2020 by the U.S. FDA  Approved on Mar 24, 2021 by NMPA |
|  | Selpercatinib | Locally advanced or metastatic RET-positive NSCLC | Approved on Mar 8, 2020 by the U.S. FDA |
| *NTRK* | Larotrectinib | TRK fusion-positive solid tumors | Approved on Nov 26, 2018 by the U.S. FDA  Approved on Apr 13, 2022 by NMPA |
|  | Entrectinib | TRK fusion-positive solid tumors | Approved on Aug 15, 2019 by the U.S. FDA |
|  |  | ROS1-positive NSCLC | Approved on Aug 15, 2019 by the U.S. FDA  Approved on July 29, 2022 by NMPA |
| *BRAF* | Dabrafenib | Combined with Trametinib for BRAF V600E-positive NSCLC | Approved on June 22, 2017 by the U.S. FDA  Approved on Dec 19, 2019 by NMPA |
| *MEK* | Trametinib | Combined with Dabrafenib for BRAF V600E-positive NSCLC | Approved on June 22, 2017 by the U.S. FDA  Approved on Dec 19, 2019 by NMPA |
| *FGFR* | Erdafitinib | FGFR-positive bladder cancer | Approved on Apr 12, 2019 by the U.S. FDA  has no NSCLC indication yet |
|  | Infigratinib | FGFR-positive Cholangiocarcinoma | Approved on May 28, 2021 by the U.S. FDA  Has no NSCLC indication yet |

BRAF: V-raf murine sarcoma viral oncogene homolog B1; FGFR: Fibroblast growth factor receptor; KRAS: Kirsten rat sarcoma viral oncogene; MEK: Mitogen-activated protein kinase; NMPA: National Medical Product Administration; NSCLC: Non-small cell lung cancer; NTRK: Neurotrophin receptor kinase; RET: RET proto-oncogene; ROS: ROS proto-oncogene 1; TKI: Tyrosine kinase inhibitor; TRK: Trophin receptor kinase; U.S. FDA: United States Food and Drug Administration.
